# Biosynthesized Silver Nanoparticles From *Bacillus stercoris* 2AT10: Synthesis, Characterization, and Antimicrobial Activity Against Paronychia‐Associated Microorganisms

**DOI:** 10.1155/ijm/8867795

**Published:** 2026-08-12

**Authors:** Camilla A. S. Valença, André L. S. Santos, Eliana B. Souto, Patrícia Severino, Sona Jain

**Affiliations:** ^1^ Postgraduate Program in Industrial Biotechnology, Tiradentes University, Aracaju, Sergipe, Brazil, unit.br; ^2^ Department of General Microbiology, Paulo de Góes Institute of Microbiology, Federal University of Rio de Janeiro (UFRJ), Rio de Janeiro, Brazil, ufrj.br; ^3^ UCD School of Chemical and Bioprocess Engineering, University College Dublin, Belfield, Dublin 4, Ireland, ucd.ie; ^4^ Postgraduate Program in Biology of Infectious and Parasitic Agents, Federal University of Sergipe, São Cristóvão, Sergipe, Brazil, ufs.br; ^5^ Postgraduate Program in Biotechnology (RENORBIO), Federal University of Sergipe, São Cristóvão, Sergipe, Brazil, ufs.br

**Keywords:** antimicrobial activity, *Bacillus stercoris*, green nanotechnology, paronychia, silver nanoparticles

## Abstract

Paronychia represents an underexplored clinical condition with limited alternatives to conventional antimicrobial therapies, which are increasingly compromised by antibiotic resistance and toxicity. Here, we report for the first time the extracellular biosynthesis of silver nanoparticles (BAgNPs) using *Bacillus stercoris* 2AT10, a marine‐derived bacterium not previously exploited for nanoparticle production. By integrating green nanotechnology with genome mining approaches, we investigated the molecular basis underlying nanoparticle formation and evaluated the potential of BAgNPs against pathogens associated with paronychia. Physicochemical characterization by ultraviolet–visible (UV‐Vis) spectroscopy confirmed the presence of particles, with mean sizes ranging from 47 to 87 nm measured by dynamic light scattering (DLS). Their spherical shape was confirmed by transmission electron microscopy (TEM), whereas Fourier‐transform infrared (FTIR) spectroscopy suggested the presence of hydroxyl, amide, and nitrogen‐containing groups possibly associated with reducing or capping action. Genes encoding nitrate reductases, laccases, and bioactive secondary metabolites, such as bacillibactin, subtilosin A, bacillaene, bacilysin and fengycin, that may be involved in silver ion reduction and nanoparticle stabilization were identified by genome annotation using eggNOG‐mapper and antiSMASH. The biosynthesized AgNPs exhibited potent broad‐spectrum antimicrobial activity against paronychia‐associated pathogens, notably with inhibition zones of 25.8 mm for *Staphylococcus aureus* and of 23.2 mm for *Pseudomonas aeruginosa*. The Hen′s Egg Test on the Chorioallantoic Membrane (HET‐CAM) assay showed the nonirritating profile of the biosynthesized particles (irritation index ≤ 1), supporting further investigation of their suitability for topical applications, pending additional biocompatibility studies. Collectively, these findings establish *B. stercoris*–derived BAgNPs as a promising green nanotechnology platform to address the unmet need for safe and effective prophylactic agents against bacteria associated with paronychia, with additional potential as antimicrobial preservatives for dermocosmetic formulations.

## 1. Introduction

The skin plays a fundamental role as the first defensive barrier against opportunistic pathogens. When the periungual tissue is disrupted by trauma, chronic moisture exposure, manipulation of the cuticle, or other local injuries, the proximal and lateral nail folds become susceptible to infection, such as paronychia. Acute paronychia is most frequently associated with bacteria such as *Staphylococcus aureus*, *Staphylococcus epidermidis*, *Pseudomonas aeruginosa*, and *Candida albicans* in chronic cases [1–3]. This microbiological diversity, together with the persistence and recurrence observed in some cases, supports the search for topical antimicrobial approaches with broad‐spectrum activity and a favorable local safety profile.

Current management of paronychia depends on the clinical severity and etiology and may include drainage, local care, topical agents, and systemic antibiotics in selected purulent or spreading infections [3]. However, conventional antimicrobial treatment can be limited by reduced penetration into infected periungual tissues, the participation of biofilm‐forming microorganisms, and the increasing prevalence of antibiotic‐resistant bacteria, especially *S. aureus*. These limitations are relevant for anatomically localized infections, such as paronychia, where a topical delivery system able to act against Gram‐positive bacteria, Gram‐negative bacteria, and fungi would be particularly attractive. Many of the currently available products are formulated with natural plant oils containing active compounds, as well as oral antimicrobial drugs like amoxicillin–clavulanate or clindamycin [3].

Nanotechnology has played a pivotal role in improving the efficient delivery of antimicrobial compounds to affected areas. Metallic nanoparticles can be produced using a variety of methods, and innovative approaches in nanoparticle synthesis (such as those employing natural extracts) show promise by eliminating the need of chemical reducing agents [4–6]. One such biosynthesis technique involves bacterial extracts containing metabolites that reduce and stabilize nanoparticles [7]. Research has demonstrated that biosynthesized silver nanoparticles (AgNPs) exhibit superior antimicrobial activity, lower toxicity in biomedical uses, and reduced synthesis times, leading to a lower risk of environmental impact compared to chemically synthesized counterparts [8–10]. AgNPs may disrupt microbial membranes, promote the production of reactive oxygen species (ROS), interfere with proteins and nucleic acids, release bioactive Ag^+^ ions, and impair quorum sensing and biofilm formation, thereby acting through multiple mechanisms that are less dependent on a single conventional drug target [11]. These properties have motivated intense interest in environmentally friendlier production routes, including biogenic synthesis using microorganisms or their metabolites. In contrast to conventional chemical synthesis, microbial biosynthesis can reduce the need of harsh reducing agents and may simultaneously provide capping biomolecules that contribute to colloidal stability and biological activity [10, 11].

The antimicrobial potential of bacterial extracts rich in metabolites has been widely studied, particularly in bacteria isolated from poorly explored environments, with the goal of identifying novel molecules with proven efficacy [12]. The genus *Bacillus*, commonly associated with soil sediments and marine environments, is known for producing a diverse range of secondary metabolites and enzymes with antifungal, antibacterial, and antioxidant properties.

Members of the genus *Bacillus* are particularly relevant for AgNP biosynthesis. *Bacillus* spp. are metabolically versatile bacteria that secrete enzymes, peptides, proteins, and other secondary metabolites capable of reducing Ag^+^ to elemental silver and stabilizing the resulting nanoparticles. Recent studies indicate that extracellular synthesis using *Bacillus* culture supernatants is especially attractive because it simplifies downstream recovery and avoids the need to disrupt biomass [9, 11, 13]. In *Bacillus*‐mediated systems, nitrate reductase– and oxidoreductase‐related pathways have been implicated in silver ion reduction, while proteins and other secreted biomolecules appear to function as both reducing and capping agents [11, 13]. The resulting particles are most often predominantly spherical, commonly exhibit surface plasmon resonance (SPR) bands in the 410–440 nm region, and display antimicrobial activity against clinically relevant bacteria and fungi, although size, dispersity, stability, and potency vary substantially according to strain and synthesis conditions [5, 8, 9, 13].

*Bacillus*‐derived biogenic AgNPs have already shown activity against several microorganisms that are directly relevant to paronychia. AgNPs synthesized with *Bacillus subtilis* have shown inhibitory activity against *S. aureus*, methicillin‐resistant *S. aureus* (MRSA), *S. epidermidis*, and *C. albicans* [4, 9], while *Bacillus pumilus*–mediated AgNPs have demonstrated potent activity against *S. aureus* and *P. aeruginosa* together with high synthesis efficiency and prolonged colloidal stability [13]. More recently, AgNPs generated by *Bacillus lentus* and *Bacillus velezensis* have reinforced the view that *Bacillus* metabolites can support reproducible nanoparticle formation with antibacterial and, in some cases, antibiofilm effects [5, 8]. However, a major limitation in the field is that nanoparticle formation and function are strongly influenced by the biochemical composition of the biological extract used for synthesis. Because each *Bacillus* strain secretes a distinct repertoire of enzymes, peptides, and secondary metabolites, data obtained with *B. subtilis*, *B. pumilus*, *B. lentus*, or *B. velezensis* do not establish that the same synthesis efficiency, capping profile, particle characteristics, or antimicrobial behavior will occur with *Bacillus stercoris*.

Recent reviews emphasize persistent challenges in biogenic AgNP research, including incomplete identification of the molecules responsible for reduction and stabilization, sensitivity of synthesis outcomes to culture conditions, batch‐to‐batch reproducibility issues, limited toxicological characterization, and the scarcity of application‐oriented studies focused on clinically relevant targets [11]. These limitations are particularly important for *B. stercoris* 2AT10.

Our previous work demonstrated that a *B. stercoris* 2AT10 isolate from Brazilian coastal sand harbors biosynthetic gene clusters responsible for the possible production of subtilosin A, bacilysin, bacillaene, and a lactococcin‐like bacteriocin [14]. In the in vitro tests, its cell‐free supernatant showed broad‐spectrum activity against *S. aureus*, including MRSA strains, without causing irritation in ex vivo assays [14]. Those findings indicate that *B. stercoris* 2AT10 produces extracellular metabolites of antimicrobial interest and suggest that its secretome may contain compounds capable of participating in metal ion reduction and nanoparticle stabilization. However, no published study has established whether *B. stercoris*—including strain 2AT10—can biosynthesize AgNPs extracellularly, which metabolites are involved in that process, or whether the resulting nanoparticles retain physicochemical features compatible with antimicrobial application. Thus, although the strain is already recognized as a promising antimicrobial producer, its value as a biogenic nanomaterial platform remains to be explored.

The biosynthetic potential of peptide‐ and polyketide‐associated antimicrobial compounds raises the possibility that the same extracellular milieu used for nanoparticle synthesis may influence nucleation, growth, capping, and biological performance. For *B. stercoris* 2AT10, key parameters (e.g., whether silver ion reduction occurs efficiently under extracellular conditions, what synthesis conditions are suitable, what particle size and morphology are obtained, which biomolecular functional groups remain associated with the nanoparticle surface, how stable the colloid is, and whether any antimicrobial advantage emerges against clinically relevant pathogens) have not been defined yet. The safety profile of the nanoparticle formulation cannot be inferred from the non‐irritating (NI) nature of the crude cell‐free supernatant, because nanosilver introduces additional determinants of biological interaction, including size‐dependent penetration and oxidative stress–related effects. Paronychia‐associated microorganisms provide an appropriate and clinically meaningful test panel, as they represent a mixed group of bacterial and fungal pathogens frequently implicated in periungual infection, allowing assessment of whether a single biogenic nanomaterial can act across distinct cell envelope architectures and microbial physiologies [2, 3]. *S. aureus*, including MRSA, is a key player in acute paronychia and is often responsible for treatment failures in skin and soft‐tissue infections. This makes it a primary target for new antimicrobial strategies. *Staphylococcus epidermidis*, *P. aeruginosa* and *C. albicans* are also relevant because of their capacity to persist in moist periungual environments and to participate in chronic, recurrent, or polymicrobial infections.

This study describes for the first time the biosynthesis of AgNPs using *B. stercoris* extract (BAgNPs) containing products from bacterial metabolism. The potential activity against microorganisms associated with paronychia and irritation index were analyzed in vitro and ex vivo, respectively, and bioinformatics tools were used to predict metabolites possibly associated with the reducing and antimicrobial activity of bacterial extracts. The localized nature of paronychia makes it a plausible setting for future topical applications of AgNP‐containing formulations, provided that antimicrobial efficacy is balanced by acceptable irritation and safety profiles.

## 2. Materials and Methods

### 2.1. Biosynthesis of Silver Nanoparticles

The bacterial strain *B. stercoris* 2AT10 used in this work was previously isolated by us [14] from the beach sand (Praia da Atalaia, Aracaju‐SE/Brazil) and grown in nutrient broth (Kasvi, Pinhais, Paraná, Brazil) at 37°C for 48 h in static conditions. The bacterial extract used for biosynthesis was obtained by centrifugation (XHettich Lab Technology, Universal 320 R, Tuttlingen, Germany) at 10,000 rpm for 25 min at 4°C of the broth. The *B. stercoris* extract obtained after centrifugation was then mixed with previously made silver nitrate (AgNO_3_) solutions at concentrations of 1, 3, and 5 mM, prepared using deionized water (Milli‐Q water, Merck Millipore, Burlington, Massachusetts, United States). The mixed proportions (presented in Table 1) were incubated for 24 h at room temperature, under magnetic agitation at 180 rpm. Nutrient broth was used as the negative control.

**Table 1:** Composition of the biosynthesized silver nanoparticles (BAgNPs) prepared using different concentrations of AgNO_3_ and *Bacillus stercoris* 2AT10 cell‐free extract, with the final reaction volume adjusted to 20 mL for all formulations.

BAgNPs	AgNO_3_ solution (mL)			*Bacillus stercoris* 2AT10 extract (mL)
	1 mM	3 mM	5 mM	
BAgNP1	2	—	—	18
BAgNP3	—	6	—	14
BAgNP5	—	—	10	10

### 2.2. UV‐Vis Spectroscopy

Analyses to detect the formation of BAgNPs were conducted using a Hach spectrophotometer (DR 5000, Hach Company, Loveland, Colorado, United States), scanning over a range of 190–800 nm. Milli‐Q water was used as a blank reference. The plasmon bands of the AgNPs reported in the literature to appear between 380 and 450 nm [15] were used as a reference.

### 2.3. Dynamic Light Scattering (DLS)

The average size (*Z*‐Ave) and polydispersity index (PDI) of the particles synthesized with different concentrations of AgNO_3_ (1, 3, and 5 mM) were determined by DLS in a Malvern Zetasizer (Nano Z‐S, Malvern Instruments, Worcestershire, United Kingdom). The analyses were carried out at an angle of 90° at 25°C and using a 633 mW He‐Ne laser with a wavenumber of 632.8 nm. The measurements were performed considering the silver refractive index 1.390, particle absorption coefficient 0.002, water refractive index 1.330, viscosity 0.8872 CP, equilibrium time 2 min, and temperature 25°C [16]. The results are the average ± standard deviation (SD) of 10 runs per measurement of a total of three readings (*n* = 30).

### 2.4. Fourier‐Transform Infrared (FTIR) Vibrational Spectroscopy

FTIR analyses were carried out using a Vertex 70 V Bruker spectrophotometer (Billerica, Massachusetts, United States) coupled to a zinc selenide (ZnSe) diamond/crystal and attenuated total reflection (ATR) device (Agilent Cary 630, Agilent Technologies, Santa Clara, California, United States). About 5 *μ*L of the liquid sample was deposited on the surface of the ATR crystal in ZnSe spectral scale of 400–4000 cm^−1^ and a resolution of 4 cm^−1^ and processed for automatic data acquisition by Origin 8.5 software [9].

### 2.5. Transmission Electron Microscopy

For the analysis of particle morphology, the transmission electron microscope (JEOL JEM 100CX II, 100KV, Akishima, Tokyo, Japan) was used. A drop of each biosynthesized AgNP (BAgNP1, BAgNP3, and BAgNP5) was deposited on a grid, and the excess sample was removed using filter paper. The sample was fixed using either a 1% solution in 45% ethanol or a 2% solution in H_2_ [17].

### 2.6. Antimicrobial Activity

Antimicrobial activity of the BAgNPs was analyzed using the agar diffusion method [18], with 40 *μ*L of BAgNPs in each well. The pathogenic indicators used were *Escherichia coli* (ATCC 25922), *Staphylococcus aureus* (ATCC 25923), *Pseudomonas aeruginosa* (ATCC 27853), *Candida albicans* (ATCC 90028), *Candida tropicalis* (ATCC 750), and *Candida parapsilosis* (ATCC 22019), all obtained from ATCC (Manassas, Virginia, United States). Pure bacterial extract (40 µL/well) was used as control to compare the synergistic effect between BAgNPs and the metabolic content.

### 2.7. Metabolite Prediction

The genome‐wide functional annotation tool eggNOG‐mapper v2 (http://eggnog-mapper.embl.de/) [19] was used to identify functional genes, metabolic pathways and genes potentially associated with silver nitrate reduction. Biosynthetic gene clusters involved in the production of secondary metabolites were predicted using antiSMASH (https://antismash.secondarymetabolites.org). Subsequently, the *B. stercoris* 2AT10 (Gen Bank IDGCA_041765565.1, RefSeq ID GCF_041765565.1; Accession Number: JBEMBP000000000) locus tags were used to confirm the functional annotations of the predicted genes in UniProt (https://www.uniprot.org/).

### 2.8. Hen′s Egg Test on Chorioallantoic Membrane (HET‐CAM)

To qualitatively evaluate the irritation potential of BAgNPs, the HET‐CAM was performed using fertilized chicken eggs (Asa Branca, São Cristóvão, Sergipe, Brazil). Sodium hydroxide solution 1 M (NaOH, analytical grade, Neon Comercial, São Paulo, Brazil) and sodium chloride solution 0.9% (NaCl, analytical grade, Neon Comercial, São Paulo, Brazil) were both prepared with Milli‐Q water. Both solutions and *B. stercoris* 2AT10 extract were used as controls. All experiments were conducted in triplicate. Vascular responses, including hyperemia (H), hemorrhage/vessel lysis (L), and coagulation (C), were monitored and documented by camera over a 5‐min observation period. The irritation index score (IIS) was assigned according to the time of onset of each vascular response, as described in Table 2.

**Table 2:** Classification criteria of the irritation phenomena observed in the chorioallantoic membrane (hyperemia, hemorrhage or vessel lysis, and coagulation) as a function of time, used to determine the irritation index score (IIS)

Time	Hyperemia	Hemorrhage	Coagulation/opacity
30 s	5	7	9
30 s to 2 min	3	5	7
2–5 min	1	3	5
No reaction	0	0	0

The IIS was calculated using the following equation:

IIS=301−H×5300+301−L×7300+301−C×9300/

where *H*, *L*, and *C* represent the time‐weighted scores for hyperemia, hemorrhage/vessel lysis, and coagulation, respectively. When no reaction was observed within the corresponding time, the variable was set to 301. The IIS values were used to classify the irritation potential as follows: *non-irritant* (0 ≤ IIS ≤ 0.9), *slightly irritant* (1 ≤ IIS < 4.9), *moderately irritant* (5 ≤ IIS ≤ 9.9), and *severely irritant* (10 ≤ IIS ≤ 21) [20, 21].

### 2.9. Statistical Analyses

All experiments were conducted independently and, at least, in triplicate, and the results were expressed as means ± SD of biological replicates. For absorbance measurements, the values were normalized by the control condition. The differences between groups were verified using one‐way (1‐way) ANOVA with GraphPad Prism (GraphPad Software, San Diego, California, United States), followed by Tukey′s HSD post hoc test (*α* = 0.05) to compare antimicrobial activity across treatments. Irritation indice scores (IIS) from the HET‐CAM assay were analyzed using the Kruskal–Wallis test. Post hoc pairwise comparisons between treatments and controls were performed using the Mann–Whitney *U* test. Statistical significance was set at *α* = 0.05.

## 3. Results

### 3.1. BAgNP Synthesis and Characterization

**Figure 1:**
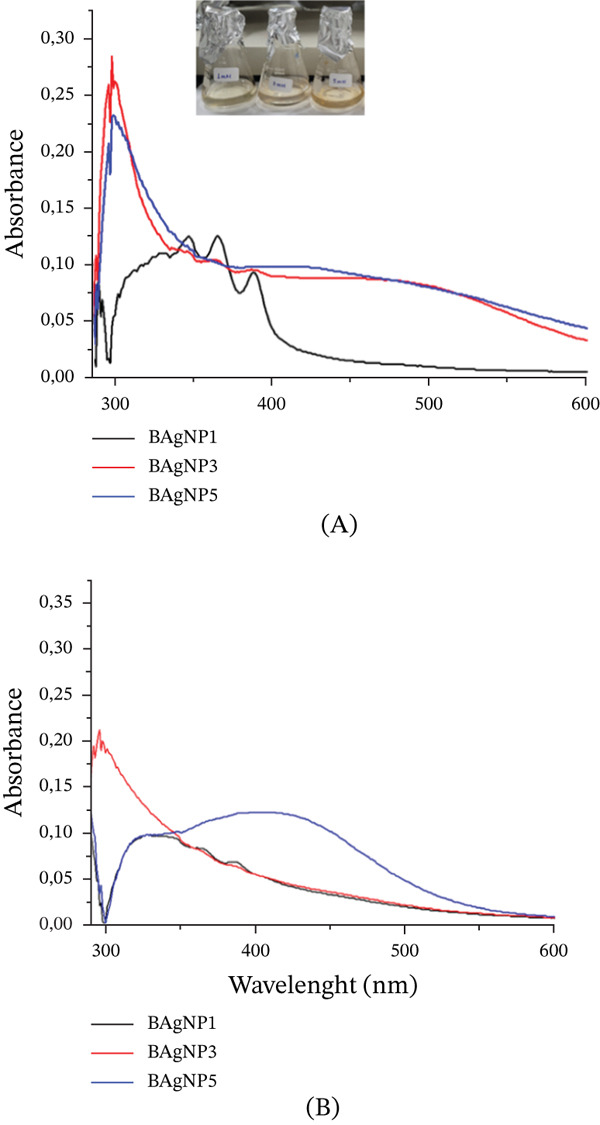
(A) UV‐Vis spectra of BAgNPs biosynthesized from the *B. stercoris* 2AT10 extract mixed with silver nitrate solutions at 1, 3, and 5 mM. Colorimetric changes of the solutions are displayed in insert. Due to the complex molecular composition of the extract, all the absorption bands show noise detected during the readings. (B) UV‐Vis spectra of biosynthesized BAgNP1, BAgNP3, and BAgNP5 after centrifugation steps aimed at removing interfering substances.

After mixing the *B. stercoris* extract with each of the three AgNO_3_ solutions at 1, 3, and 5 mM, respectively, a gradual change in color was observed (Figure 1A, insert), with an increase in intensity proportional to the increase in silver concentration. UV‐Vis spectroscopy showed an increase in absorbance in the region between 280 and 310 nm (Figure 1). No significant color change was observed for the control containing only the nutrient broth culture medium and AgNO_3_ (1, 3, and 5 mM) solution. After the centrifugation step, the BAgNP5 showed an absorbance peak in the range of 400–410 nm, while BAgNP1 and BAgNP3 showed absorption peak in the range of 300‐310 and 300‐340 nm, respectively.

A concentration‐dependent increase in nanoparticle size was observed (Table 3) when comparing BAgNP1 and BAgNP5, with *Z*‐Ave values rising from 47.18 nm (BAgNP3) to 87.44 nm (BAgNP5). PDI values decreased with increasing silver concentration (0.500 for BAgNP1 to 0.286 for BAgNP5), indicating improved size uniformity at higher molarities.

**Table 3:** Mean particle size (Z‐Ave) and polydispersity index (PDI) of the BAgNPs recorded by dynamic light scattering.

BAgNPs	*Z* − *A* *v* *e* ± *S* *D*(nm)	*P* *D* *I* ± *S* *D*
BAgNP1	48.47 ± 3.07	0.500 ± 0.041
BAgNP3	47.18 ± 0.68	0.455 ± 0.009
BAgNP5	87.44 ± 2.66	0.286 ± 0.013

### 3.2. Transmission Electron Microscopy

**Figure 2:**
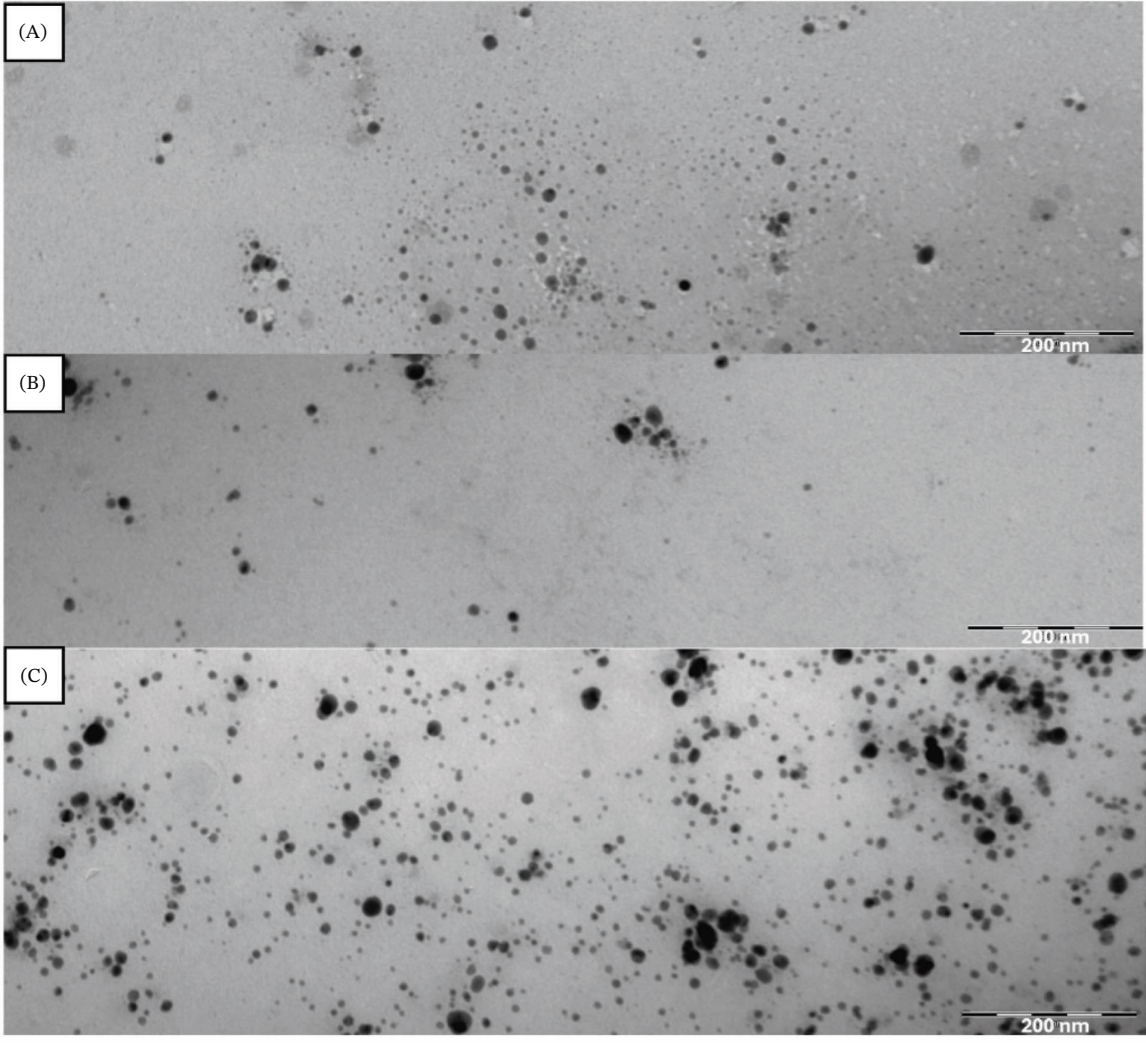
Transmission Electron Microscopy (TEM) micrographs of biosynthesized silver nanoparticles prepared with 1 mM (A), 3 mM (B), and 5 mM (C) AgNO_3_ solutions. All formulations consisted predominantly of spherical nanoparticles. BAgNP5 displayed a higher particle density and greater size heterogeneity than BAgNP1 and BAgNP3. Scale bars = 200 nm.

The presence of individual nanoparticles was confirmed by the images obtained from TEM (Figure 2). TEM imaging confirmed spherical morphology across the three developed formulations, although the 5 mM sample (BAgNP5) exhibited greater size heterogeneity despite lower PDI values.

### 3.3. FTIR Spectroscopy

**Figure 3:**
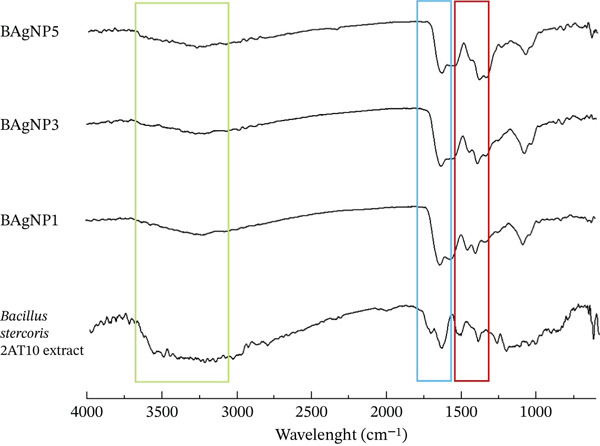
FTIR spectra of BAgNP1, BAgNP3, BAgNP5 and of the *B. stercoris* 2AT10 extract. Spectra of the extract and the silver nanoparticles were found similar, highlighting the presence of hydroxyl groups marked in green (‐OH), amide groups marked in blue (C=O), and nitrogen‐containing groups marked in red (‐NH).

Common stretching vibrations were found in FTIR analysis for all nanoparticle batches (Figure 3) (i.e., 3221.12 [BAgNP1], 3275.13 [BAgNP3], and 3271.27 cm^−1^ [BAgNP5]), corresponding to hydroxyl ‐OH bonds, which may be associated with the reduction of BAgNPs in an aqueous medium [9, 22]. Bands at 1566.20 cm^−1^ (BAgNP1), 1564.27 cm^−1^ (BAgNP3), and 1554.63 cm^−1^ (BAgNP5) were assigned to amide (N–H/C=O) vibrations of proteins [23]. In the *B. stercoris* 2AT10 extract, a band at ~3250 cm^−1^ confirms O–H/N–H stretching, and the band at 1637.56 cm^−1^ is consistent with C=O of amides, suggesting the presence of proteins acting as reducing and/or capping agents [5, 24, 25].

### 3.4. Antimicrobial Potential

The results of the antimicrobial activity of BAgNPs are depicted in Table 4. All BAgNP1,BAgNP3 and BAgNP5 inhibited the growth of the tested microorganisms. For *S. aureus*, significant differences were detected among treatments (*p* < 0.001), with the greatest inhibition observed with BAgNP5. However, a nonlinear dose–response pattern was evident, as the BAgNP1 treatment was more effective than the BAgNP3 treatment. Significant differences were also found for *P. aeruginosa* (*p* < 0.01), with growth inhibition increasing from BAgNP1 to BAgNP5. For *E. coli*, antimicrobial activity differed significantly among treatments (*p* < 0.01), with the highest inhibition observed with BAgNP1 treatment and a decline in activity at higher concentrations. Significant treatment effects were also observed for *Candida albicans* (*p* < 0.05) and *Candida tropicalis* (*p* < 0.01), with maximum growth inhibition recorded with BAgNP1 and BAgNP3, respectively. In contrast, no significant concentration‐dependent differences were observed for *C. parapsilosis* (*p* > 0.05).

**Table 4:** Antimicrobial activity (expressed as mm of inhibition zone) of BAgNP1, BAgNP3, and BAgNP5 against *Staphylococcus aureus*, *Pseudomonas aeruginosa*, *Escherichia coli*, *Candida albicans*, *Candida tropicalis*, and *Candida parapsilosis*. *B. stercoris* 2AT10 extract was used as a control. Values are expressed as mean ± standard deviation (SD) (*n* = 6). Different lowercase letters within the same row indicate significant differences among BAgNPs, determined by one‐way ANOVA followed by Tukey′s multiple comparison test (*p* < 0.05).

Microorganism	Inhibition zone (mm)			
	*B. stercoris* 2AT10 extract	BAgNP1	BAgNP3	BAgNP5
*S. aureus* (ATCC 25923)	10.0 ± 0.1	18.4 ± 0.1^b^	12.2 ± 0.1^c^	25.8 ± 0.2^a^
*P. aeruginosa* (ATCC 27853)	5.7 ± 0.3	8.0 ± 0.1^c^	9.4 ± 0.2^b^	23.2 ± 0.1^a^
*E. coli* (ATCC 25922)	4.3 ± 0.2	11.4 ± 0.1^a^	9.1 ± 0.1^b^	8.5 ± 0.1^c^
*C. albicans* (ATCC 90028)	No inhibition observed	15.0 ± 0.1^a^	8.5 ± 0.2^c^	10.5 ± 0.1^b^
*C. tropicalis* (ATCC 750)		12.0 ± 0.1^b^	6.2 ± 0.1^c^	20.0 ± 0.1^a^
*C. parapsilosis* (ATCC 22019)		13.0 ± 0.2^a^	13.3 ± 0.1^b^	14.7 ± 0.3^a^

### 3.5. Bacterial Metabolites Predicted in the *B. stercoris* 2AT10 Genome

Genes encoding enzymes previously associated with redox reactions and nanoparticle biosynthesis in other microorganisms are listed in Table 5. The eggNOG‐mapper analysis revealed the presence of genes coding for nitrate and nitrite reductase enzymes as well as other reductase enzymes that have been reported in the literature as potential contributors to metal reduction processes. Genes encoding enzymes involved in *γ*‐polyglutamic acid (*γ*‐PGA) biosynthesis and tripeptide metabolism were also identified, including the PGA synthase capB and the tripeptidase pepT.

**Table 5:** Genes present in the genome of *B. stercoris* 2AT10 coding possible bacterial enzymes related to silver nitrate reduction and silver nanoparticle formation.

Gene	Description	Function	References
cotA	Laccase	Multicopper oxidase that catalyzes the oxidation of a variety of substrates, including phenolic and nonphenolic compounds.	[26]
narI	Nitrate reductase (gamma chain)	The gamma chain is a membrane‐embedded heme iron unit resembling Cytochrome b, which transfers electrons from quinones to the beta subunit.	[27]
narJ	Nitrate reductase	Chaperone required for proper molybdenum cofactor insertion and final assembly of the membrane‐bound respiratory nitrate reductase.	
narH	Nitrate reductase (beta chain)	The beta chain is an electron transfer unit containing four cysteine clusters involved in the formation of iron–sulfur centers.	
narG	Nitrate reductase (alpha chain)	The alpha chain is the actual site of nitrate reduction.	
yodC	Nitroreductase	Putative nitroreductase that may contribute to the degradation of aromatic compounds.	[28]
ydfN			[29]
yfhC			[28]
ydgI			[28]
frp		Reduces FMNH_2_ to FMN, with NADH or NADPH as reductant.	[30]
nasB	Assimilatory nitrate reductase (electron transfer subunit)	Required for nitrate assimilation.	[31]
nasC	Assimilatory nitrate reductase catalytic subunit	A key enzyme that is involved in the first step of nitrate assimilation in plants, fungi, and bacteria.	[28]
nasD	Assimilatory ferredoxin‐dependent nitrite reductase	Catalyzes the reduction of nitrite to ammonium in the nitrate assimilation pathway, using ferredoxin as the electron donor.	[28]
nasE	Nitrite reductase	Required for nitrite assimilation and for the activity of the reductase.	[29]
capB	PGA synthase	Catalyzes the biosynthesis of PGA (gamma‐polyglutamic acid) from L‐glutamate.	[30]
pepT	Peptidase T	Cleaves the *N*‐terminal amino acid of tripeptides.	[32]

### 3.6. HET‐CAM

**Figure 4:**
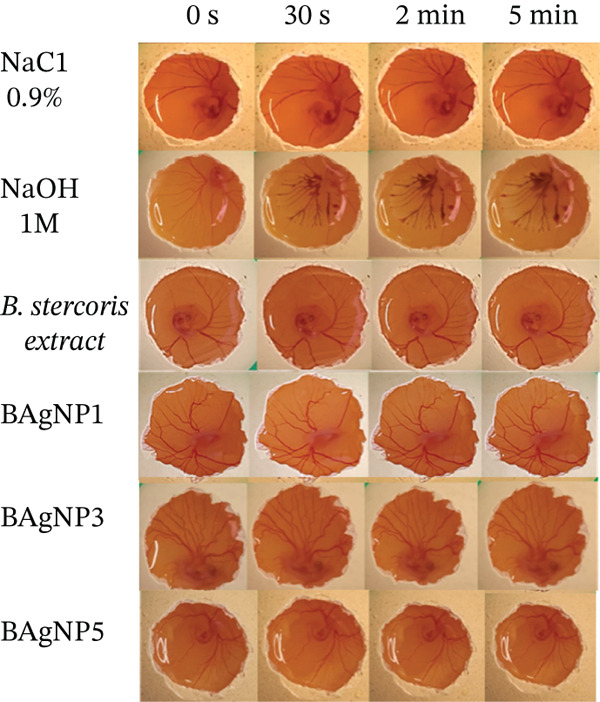
Images of chorioallantoic membranes at times 0 s, 30 s, 2 min, and 5 min exposed to *B. stercoris* extract, BAgNP1, BAgNP3, and BAgNP5 and the respective positive NaOH (1 M) and negative NaCl (0.9%) controls. The Kruskal–Wallis test revealed a statistically significant difference in irritation indices among groups (*H* = 15.75, df = 5, *p* < 0.001) (Table 6). Post hoc Mann–Whitney *U* tests demonstrated that all BAgNPs (1, 3, and 5 mM) and bacterial extract exhibited irritation profiles statistically like the negative control (NaCl 0.9%, *p* > 0.05) and are significantly different from the positive control (NaOH 1 M, *p* < 0.001). However, BAgNP5 showed mild hyperemia at 2 min (IIS = 1).

As shown in Figure 4, *B. stercoris* 2AT10 extract and BAgNP1 and BAgNP3 did not induce vascularization or any alterations in the membrane structure. In contrast, CAM exposed to BAgNP5 exhibited slight hyperemia (IIS = 3.02); however, this response remained within the NI score, indicating low irritation potential and supporting further investigation of these particles for topical applications.

Hyperemia is characterized by the appearance or increased visibility of capillary vessels that were absent or not apparent prior to exposure and, according to the IIS obtained, is considered NI. Membranes exposed to the positive control exhibited intense bleeding within the first 30 s of exposure, whereas the negative control did not cause any observable membrane alterations (Figure 4). Table 6 shows the results of reaction time, IIS, and their respective classification in the HET‐CAM.

**Table 6:** Reaction time, irritation index score (IIS) and their respective classification in the HET‐CAM test of the *B. stercoris* 2AT10 extract, BAgNP1, BAgNP3 and BAgNP5, and the positive and negative controls. All samples were classified as non‐irritating based on the IIS.

Samples	HT1	HT2	CT	Irritation index score	Classification
*B. stercoris* 2AT10 extract	0	0	0	0	NI
BAgNP1	0	0	0	0	NI
BAgNP3	0	0	0	0	NI
BAgNP5	2 min	0	0	1	NI/SI
NaOH 1 M	30 s	2 min	5 min	21	SVI
NaCl 0.9%	0	0	0	0	NI/SI

Abbreviations: CT, coagulation time; HT1, hyperemia time; HT2, hemorrhage/vessel lysis time; NI, non‐irritating; SI, slightly irritating; SVI, severely irritating.

## 4. Discussion

The biosynthesis of AgNPs using bacterial extract represents a more advantageous and less time‐consuming alternative to chemical synthesis. In this study, UV‐Vis spectroscopy revealed absorption peaks at 280–310 nm across all BAgNPs (Figure 1A). These results diverge from the typical plasmon resonance range of 408–460 nm reported for other *Bacillus*‐synthesized AgNPs [33, 34]. After centrifugation, a shift in the band to 410 nm was observed only for BAgNP5 (Figure 1B). This phenomenon reflects the complex molecular composition of *B. stercoris* extract, where secondary metabolites interact to form nanoparticles and alter the surface plasmon band [35].

The absence of a clearly defined SPR band in BAgNP1 and BAgNP3 may indicate lower nanoparticle yield, smaller metallic core sizes (also recorded by TEM), masking effects caused by biomolecules present in the bacterial extract, and/or incomplete reduction of Ag^+^ ions. In addition, absorption in the 280–310 nm region is not characteristic of AgNP SPR and is more likely associated with proteins, peptides, aromatic amino acids, and other biomolecular constituents present in the bacterial extract. Differences in the optical properties of the BAgNPs were observed according to the AgNO_3_ concentration used during synthesis. The multiple absorption peaks detected in BAgNP1 may reflect nanoparticle heterogeneity as well as interference from residual biomolecules. Because BAgNP1 was prepared with the lowest silver nitrate concentration, a greater proportion of biological compounds may have remained in the suspension, contributing to the persistence of spectral interference even after centrifugation. In contrast, BAgNP3 and BAgNP5 exhibited smoother absorption profiles, suggesting a more homogeneous nanoparticle population. Furthermore, the emergence of a more defined SPR band around 410 nm in BAgNP5 after centrifugation likely reflects the removal of soluble biomolecules and culture medium constituents that partially masked the nanoparticle plasmon resonance prior to purification. The broad absorption band observed for BAgNP5 may also be associated with nanoparticle aggregation and/or increased particle size resulting from the higher silver concentration used during synthesis. Therefore, UV‐Vis evidence for AgNP formation was strongest for BAgNP5, whereas confirmation of nanoparticle formation in BAgNP1 and BAgNP3 relied primarily on complementary TEM and DLS analyses. Moreover, although DLS analysis indicated the lowest PDI value for BAgNP5, TEM images revealed some degree of size heterogeneity, confirming that different characterization techniques capture distinct aspects of nanoparticle populations (Figure 2). FTIR analysis suggested the presence of nitrogen‐containing groups (‐NH) in BAgNPs and bacterial extract, which could be associated with protein or enzyme involvement in nanoparticle formation and stabilization (Figure 3).

The observed *Z*‐Ave values (47–87 nm, Figure 3) align with previous reports on *Bacillus-*mediated AgNP synthesis, where spherical nanoparticles ranging from 3 to 68 nm were produced [17, 33, 36]. Since *Z*‐Ave reflects the hydrodynamic diameter, encompassing both metallic core and hydrodynamic coating layer, actual core sizes may be smaller than what is suggested by DLS measurements [37]. The apparent discrepancy between low PDI values (0.286 for BAgNP5) and TEM‐observed size heterogeneity results from differences in measurement principles: DLS captures the size of solvated particles, while TEM reveals individual dry particle cores [37]. Studies indicate that lower silver nitrate concentrations (1–2.5 mM) produce smaller, more biologically active nanoparticles with enhanced size uniformity, desirable characteristics for antimicrobial applications [9, 38]. However, our findings showed that BAgNP5 exhibited lower PDI values, suggesting improved size homogeneity.

The antimicrobial activity of BAgNPs substantially exceeded that of bacterial extract alone, demonstrating a possible synergistic interaction between reduced silver and bioactive metabolites such as enzymes and bacteriocins present in the bacterial extract. Comparing inhibition profiles, BAgNP5 exhibited the largest zones against *S. aureus* (25.8 mm) and *P. aeruginosa* (23.2 mm), supporting a relationship between nanoparticle concentration and antimicrobial potency. These results align with the literature describing the broad‐spectrum activity of *Bacillus-*derived AgNPs. Wang et al. [39] reported significant activity against *C. albicans* using 10–30 nm spherical nanoparticles, while Xiong et al. [40] demonstrated efficacy against Gram‐positive and Gram‐negative bacteria using 100 nm particles. In another study, *C. albicans* exhibited sensitivity to biosynthesized AgNPs (minimum inhibitory concentration (MIC) 100 *μ*g/mL), followed by *S. epidermidis* (MIC 180 *μ*g/mL). The enhanced antimicrobial activity likely derives from multiple mechanisms: (i) sustained Ag^+^ ion release causing membrane disruption and oxidative stress, (ii) direct nanoparticle–cell interactions, and (iii) enzymatic stabilization that preserves lytic activity [41]. The biomolecular coating not only stabilizes nanoparticles but may also enhance cellular uptake and antimicrobial efficacy. Importantly, 1–5 mM concentrations have been reported as optimal against paronychia‐associated pathogens including *S. aureus*, *E. coli*, *P. aeruginosa*, and *C. albicans* [2, 3].

A limitation of the present study is the absence of comparative controls such as AgNO_3_ alone, chemically synthesized AgNPs, or conventional antimicrobial agents. The bacterial extract was selected as the experimental control because the primary objective was to evaluate whether nanoparticle biosynthesis enhanced the antimicrobial activity of metabolites produced by *B. stercoris* 2AT10. Notably, the biosynthesized nanoparticles exhibited a NI or slightly irritating profile in the HET‐CAM assay, suggesting favorable preliminary safety characteristics (Figure 4, Table 6). Previous studies have reported that the biological capping agents associated with biosynthesized AgNPs may reduce adverse effects compared with some chemically synthesized nanoparticles; however, direct comparisons were beyond the scope of the present study. Future investigations should include chemically synthesized AgNPs and AgNO_3_ controls to assess their relative antimicrobial efficacy and safety.

Although *B. subtilis* has been extensively studied for AgNP biosynthesis, this represents the first report using *B. stercoris*, reclassified from *B. subtilis* subsp. *stercoris* in 2019 [41]. This phylogenetic distinction warrants investigation, as metabolic differences between closely related species may influence nanoparticle properties and bioactivity.

Genome mining revealed genes encoding various metabolites potentially involved in silver reduction, including oxidoreductase enzymes, lipopeptides, and nonribosomal peptides. Notably, nitrate reductase genes were identified, consistent with previous reports implicating this enzyme class in bacterial AgNP synthesis [26, 42]. Although nitrate reductases are traditionally considered cytoplasmic, recent evidence demonstrates their extracellular secretion and direct involvement in metal reduction [43, 44]. Extracellular nitrate reductases in *Lactobacillus plantarum* cell‐free supernatant were reported to catalyze silver ion reduction to form AgNPs, which could support our hypothesis that *B. stercoris* 2AT10 might employ a similar extracellular biosynthetic pathway [44]. However, despite bioinformatics predictions in our study, experimental validation of gene expression and enzymatic activity remains necessary to establish definitive genotype–phenotype correlations.

**Figure 5:**
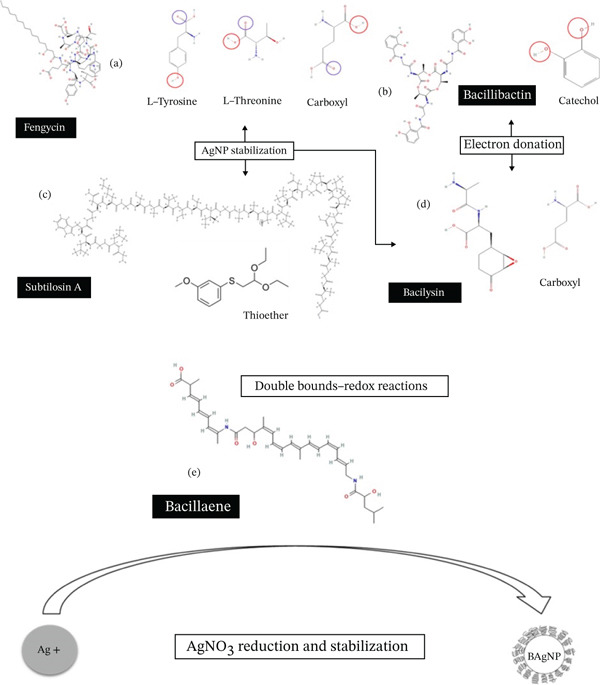
Predicted metabolites of *Bacillus stercoris* 2AT10 and their putative involvement in the extracellular biosynthesis of silver nanoparticles (AgNPs), emphasizing functional groups possibly associated with reduction and capping. (a) Fengycin, with L‐tyrosine, L‐threonine, and carboxyl groups potentially involved in nanoparticle stabilization. (b) Bacillibactin, containing catechol moieties associated with electron donation. (c) Subtilosin A, a cyclic lantipeptide whose functional groups may contribute to AgNP capping. (d) Bacilysin, a nonribosomal dipeptide containing amino and carboxyl groups that may assist in silver ion chelation. (e) Bacillaene, a polyketide containing conjugated double bonds potentially involved in redox processes.

AntiSMASH analysis predicted five key metabolites in the 2AT10 genome (bacillibactin, subtilosin A, bacillaene, fengycin, and bacilysin) with structural features potentially contributing to silver reduction and nanoparticle stabilization (Figure 5). Bacillibactin, a catechol‐containing siderophore primarily involved in Fe^3+^ chelation, can complex various metals, including silver, through similar coordination mechanisms [45], suggesting that it may contribute to silver ion reduction during nanoparticle biosynthesis. Bacillaene contains conjugated double bonds that may participate in redox reactions, whereas fengycin and subtilosin A possess functional groups that could contribute to nanoparticle stabilization and surface capping [46]. However, the direct involvement of these metabolites in Ag^+^ reduction remains to be experimentally confirmed. Although bacilysin exhibits lower reducing capacity, its polar functional groups may contribute to nanoparticle stabilization. These metabolites collectively enable both the reduction of Ag^+^ to Ag^0^ and the subsequent stabilization of nanoparticles, preventing aggregation.

HET‐CAM assay confirmed a NI profile for BAgNP1 and BAgNP3 (IIS ≤ 1), while for BAgNP5, a slightly irritation profile (IIS = 3.02). These results support a preliminary safety profile for topical applications. Previous studies have also used the HET‐CAM to assess the irritation profile of chemically synthesized AgNPs [47]. Microbial metabolites generally exhibit lower irritation compared to synthetic antimicrobials [48], further supporting the biocompatibility of *B. stercoris*–derived BAgNPs.

These properties position BAgNPs as promising candidates not only for paronychia prevention but also as antimicrobial preservatives in cosmetic formulations targeting *S. aureus* and *P. aeruginosa* contamination.

## 5. Conclusions

This study establishes *B. stercoris* 2AT10 as a novel biofactory for extracellular AgNP (BAgNP) synthesis, representing the first report of this species being applied in nanobiotechnology. By integrating genome mining with green nanotechnology, we identified key genes and biosynthetic pathways potentially involved in nanoparticle formation and stabilization, including nitrate reductases and the production of bioactive metabolites such as bacillibactin,subtilosin A, bacillaene, bacilysin and fengycin. Although these findings provide candidate mechanisms that may contribute to AgNP biosynthesis, their direct involvement remains to be experimentally validated. The biosynthesized AgNPs were predominantly spherical, with mean sizes ranging from 47 to 87 nm, and exhibited broad‐spectrum antimicrobial activity against key paronychia‐associated pathogens, including *S. aureus*, *P. aeruginosa*, and *C. albicans*. Notably, their antimicrobial efficacy was substantially greater than that of the bacterial extract alone. Furthermore, the nanoparticles exhibited no signs of irritation in the HET‐CAM assay, supporting further investigation of their suitability for topical applications.

Collectively, these findings highlight BAgNPs as a promising green nanotechnology platform with antimicrobial activity against paronychia‐associated microorganisms and other superficial microbial pathogens. Future studies should focus on elucidating the molecular mechanisms underlying nanoparticle biosynthesis, investigating formulation stability, assessing cytotoxicity against keratinocytes and fibroblasts, evaluating skin safety, and validating antimicrobial efficacy in vivo. In addition to their antimicrobial potential, the use of BAgNPs as antimicrobial preservatives in dermocosmetic formulations warrants further investigation, expanding their possible applications in other healthcare and cosmetic industries.

## Author Contributions

Camilla A. S. Valença, André L. S. Santos, Eliana B. Souto, Patrícia Severino, and Sona Jain contributed to the conceptualization, methodology, software analysis, validation, data curation and formal analysis of results, investigation, resources, writing (original draft preparation, review, and editing), supervision, project administration, and funding acquisition.

## Funding

This study was funded by the Conselho Nacional de Desenvolvimento Científico e Tecnológico; the Coordenação de Aperfeiçoamento de Pessoal de Nível Superior; the Fundação Carlos Chagas Filho de Amparo à Pesquisa do Estado do Rio de Janeiro; and the University College Dublin, 83965‐NP/R28429.

## Disclosure

All authors have read and agreed to the published version of the manuscript.

## Ethics Statement

This work does not raise ethical issues.

## Conflicts of Interest

The authors declare no conflicts of interest.

## Acknowledgments

The authors wish to thank the support received from Fundação Carlos Chagas Filho de Amparo à Pesquisa do Estado do Rio de Janeiro (FAPERJ), Conselho Nacional de Desenvolvimento Científico e Tecnológico (CNPq), and Coordenação de Aperfeiçoamento de Pessoal de Nível Superior (CAPES, Financial Code 001). Eliana B. Souto acknowledges the University College Dublin Obrss Research Scheme fund 2024–2027 (83965‐NP/R28429). Authors confirm that no AI tools have been used to draft any parts of the manuscript.

## Data Availability

The data that support the findings of this study are available on request from the corresponding author.
